# Oral activity of the antimalarial endoperoxide 6-(1,2,6,7-tetraoxaspiro[7.11]nonadec-4-yl)hexan-1-ol (N-251) against *Leishmania donovani* complex

**DOI:** 10.1371/journal.pntd.0007235

**Published:** 2019-03-25

**Authors:** Kofi Dadzie Kwofie, Kai Sato, Chizu Sanjoba, Akina Hino, Rieko Shimogawara, Michael Amoa-Bosompem, Irene Ayi, Daniel A. Boakye, Abraham K. Anang, Kyung-Soo Chang, Mitsuko Ohashi, Hye-Sook Kim, Nobuo Ohta, Yoshitsugu Matsumoto, Shiroh Iwanaga

**Affiliations:** 1 Section of Environmental Parasitology, Graduate School of Medical Dental Sciences, Tokyo Medical Dental University, Bunkyo-ku, Tokyo, Japan; 2 Laboratory of Molecular Immunology, Department of Animal Resource Sciences, Graduate School of Agricultural and Life Sciences, The University of Tokyo, Bunkyo-ku, Tokyo, Japan; 3 Department of Parasitology, Noguchi Memorial Institute for Medical Research, College of Health Sciences, University of Ghana, Legon, Accra, Ghana; 4 Department of Clinical Laboratory Science, College of Health Sciences, Catholic University of Pusan, Busan, Republic of Korea; 5 Division of International Infectious Disease Control, Faculty of Pharmaceutical Sciences, Graduate School of Medicine, Dentistry Pharmaceutical Sciences, Okayama University, Okayama, Japan; University of Oklahoma, UNITED STATES

## Abstract

Visceral leishmaniasis (VL) is a major problem worldwide and causes significant morbidity and mortality. Existing drugs against VL have limitations, including their invasive means of administration long duration of treatment regimens. There are also concerns regarding increasing treatment relapses as well as the identification of resistant clinical strains with the use of miltefosine, the sole oral drug for VL. There is, therefore, an urgent need for new alternative oral drugs for VL. In the present study, we show the leishmanicidal effect of a novel, oral antimalarial endoperoxide N-251. In our *In vitro* studies, N-251 selectively and specifically killed *Leishmania donovani* D10 amastigotes with no accompanying toxicity toward the host cells. In addition, N-251 exhibited comparable activities against promastigotes of *L*. *donovani* D10, as well as other *L*. *donovani* complex parasites, suggesting a wide spectrum of activity. Furthermore, even after a progressive infection was established in mice, N-251 significantly eliminated amastigotes when administered orally. Finally, N-251 suppressed granuloma formation in mice liver through parasite death. These findings indicate the therapeutic effect of N-251 as an oral drug, hence suggest N-251 to be a promising lead compound for the development of a new oral chemotherapy against VL.

## Author summary

Visceral Leishmaniasis remains a serious health problem in many developing countries with thousands of new cases recorded annually. Novel oral therapies are required as existing drugs are limited by their invasive means of administration long duration of treatment regimens. Moreover, with miltefosine as the sole oral drug, there are concerns of the eventual development of parasite resistance with its continuous use. In this study, we report on the *in vitro* and *in vivo* leishmanicidal effect of orally administered N-251 on the *Leishmania donovani* complex parasites in mice. Our results suggest that N-251 may be a potential lead compound for the development of a new oral chemotherapy against VL

## Introduction

Visceral leishmaniasis (VL), also known as kala azar, is a disease with a worldwide distribution. It is endemic in more than 62 countries, with over 90% of cases occurring in Brazil, Ethiopia, India, Somalia, South Sudan Sudan [1]. An estimated 200,000 to 400,000 new cases are reported annually [1], making VL a serious public health problem. It is caused by members of the *Leishmania donovani* complex, which consists of four species: *L*. *archibaldi*, *L*. *chagasi*, *L*. *donovani*, and *L*. *infantum*, which are distinguished by their vectors, reservoir hosts, and their associated pathology [2,3]. Parasites are transmitted to humans by the female phlebotomine sandfly. VL is typically presented as a chronic infection, characterized by irregular bouts of fever, weight loss, splenohepatomegaly, anemia, and a high mortality rate of almost 100% if left untreated [1]. At present, there is no licensed vaccine, therefore, chemotherapy remains the main form of control.

Currently, treatment for VL relies on the use of drugs such as pentavalent antimonials, paromomycin, pentamidine, and amphotericin B. Most of these drugs are, however, either limited by their invasive methods of administration and/or their long periods of high dose treatment. Miltefosine remains the sole oral anti-leishmanial drug since it was approved for use in 2002 [4–6]. Recently, the absence of alternate oral drugs has become a major concern, particularly with recent reports of increasing treatment relapses as well as the identification of miltefosine-resistant clinical strains [7–9]. There is, therefore, an urgent need for the development of novel oral chemotherapeutics as alternatives to miltefosine for the effective treatment of VL cases.

Recently, natural and synthetic endoperoxide compounds have received increasing attention due to their fast action and ability to target drug-resistant parasite strains [10–19]. This class of compound is known to be activated within host cells, as a result of the iron-mediated cleavage of the characteristic endoperoxide bridge. Moreover, as anti-infective agents, they are easily administered via the oral route. Previously, we synthesized a novel endoperoxide compound, 6-(1,2,6,7-tetraoxaspiro[7.11]nonadec-4-yl)hexan-1-ol (N-251) (S1 Fig), that possesses antimalarial [20,21**]**, anti-*Toxoplasma gondii* [22], anti-schistosomal [23–25], anti-viral [26,27] activities *in vitro* when administered to mice orally. These findings suggest that N-251 possesses a broad spectrum of anti-infective activities.

In the present study, we report on the *in vitro* leishmanicidal effects of N-251 against amastigotes of *L*. *donovani*, as well as the promastigotes of various *L*. *donovani* complex parasites from different geographical locations. In addition, we report on the *in vivo* efficacy of N-251 as an oral drug against *L*. *donovani* amastigotes. Results suggest that N-251 may be a promising lead compound for the development of a new oral chemotherapy against VL.

## Methods

### Reagents parasites

N-251 was chemically synthesized as described previously [20,21]. It was dissolved in dimethyl sulfoxide (DMSO) as 100 mM stock solutions stored at −20 °C. Miltefosine was purchased from Sigma-Aldrich (MO, USA). Medium 199 was purchased from Nissui Pharmaceuticals Co., Ltd, Japan. Dulbecco’s Modified Eagle’s Medium (DMEM) Roswell Park Memorial Institute (RPMI) medium were purchased from Sigma-Aldrich. HEPES Buffer (1 M) was purchased from MP Biomedicals, LLC (Ohio, USA). All reagents were maintained at 4°C unless otherwise stated. Five strains of *L*. *donovani* complex from different geographical locations with high VL burden (Brazil, Nepal, India, Sudan Turkey) were used in this study. These include *L*. *chagasi* PP75 (MHOM/BR/74/PP75), *L*. *donovani* D10 (MHOM/NP/03/D10), *L*. *donovani* DD8 (MHOM/IN/80/DD8), *L*. *donovani* KH (MHOM/SU/43/KH) (ATCC 30503), and *L*. *infantum* EP173. Promastigotes were cultured *in vitro* in M-199 complete medium (containing 25 mM HEPES at 25°C) supplemented with 10% heat-inactivated fetal bovine serum (Hi-FBS). For *in vitro* and *in vivo* infectivity assays, *L*. *donovani* D10 was used because it is constantly maintained in mice in our lab, therefore, highly infective. The usual procedure is to use freshly isolated parasites that have undergone 1–3 cycles of passages in M-199 complete medium to ensure high infectivity rates in our experiments.

### Ethics statement

All animal experiments were reviewed and approved by the Animal Experiment Committee at the Graduate School of Agricultural and Life Sciences, University of Tokyo (Ref. No. P16-254). The experiments were performed in accordance with the Regulations for Animal Care Use of the University of Tokyo, which are based on the Law for the Humane Treatment and Management of Animals, Stards Relating to the Care and Management of Laboratory Animals Relief of Pain (the Ministry of the Environment), Fundamental Guidelines for Proper Conduct of Animal Experiment Related Activities in Academic Research Institutions (the Ministry of Education, Culture, Sports, Science Technology) and the Guidelines for Proper Conduct of Animal Experiments (the Science Council of Japan). At the end of the experiments, the animals were euthanized by exsanguination under anesthesia with isoflurane followed by cervical dislocation.

### *In vitro* inhibition activity against intracellular amastigotes

The leishmanicidal effect of N-251 on *L*. *donovani* amastigotes was evaluated in murine macrophages. First, 4 x 10^4^ RAW 264.7 macrophage cells in 200 μl of DMEM (containing 1% penicillin/streptomycin, supplemented with 10% Hi-FBS) were seeded in the wells of an 8-well chamber slide incubated at 37 °C in 5% CO_2_ for 2 hours to allow cell attachment. Next, stationary phase *L*. *donovani* promastigotes in fresh DMEM were added to the macrophages at a ratio of 50:1 (parasites:macrophage) and incubated for 6 h at 37 °C in 5% CO_2_. During this period, the parasites invaded the macrophages and then transformed into amastigotes. Free promastigotes were subsequently removed by successive washes with DMEM. Infected macrophages were then treated with N-251 at concentrations ranging from 0.78 to 50 μM, and incubated for 24, 48 and 72 h. Miltefosine was used as a reference drug control. Infected treated macrophages were then washed with 1x phosphate-buffered saline (PBS) and fixed with methanol for 10 min. Finally, the macrophages were stained with 5% Giemsa in PBS for 25 min and observed under a light microscope. Anti-leishmanial activity was evaluated by observing 300 macrophages within each treatment group. The percentage of infected macrophages was calculated using the following formula: [(Number of infected macrophages/300 macrophages observed) x 100]. Infection index values were then calculated according to the following formula: [Percentage of infected macrophages x average number of intracellular amastigotes per infected macrophage]. Infection index values were then converted to percentage survival values relative to the untreated parasite population. IC_50_ values were eventually obtained by sigmoidal dose–response curve analysis using the scatter plot option of Microsoft Excel 2016 (Microsoft Corporation, Washington, USA) expressed as the mean of samples ± stard deviation (SD) from three independent experiments conducted in duplicates.

### Cytotoxicity evaluation selectivity index determination

The cytotoxicity of N-251 against murine macrophage cell lines was assessed using the Invitrogen alamar blue assay kit (ThermoFisher Scientific, Japan) according to the manufacturer’s instructions with modifications. Both RAW 264.7 and J774 cell lines were used in this assay. First, 5 × 10^3^ cells were seeded into each well of a 96-well plate. Varying concentrations of N-251, ranging from 0.195 μM to 200 μM, were then added to the cells and incubated for 48 h at 37 °C in 5% CO_2_. Miltefosine was used as a positive control. Next, 10% alamar blue dye was added to all wells and the plate was incubated for another 24 h in darkness. After a total of 72 h, fluorescence intensity was measured at a wavelength of 600 nm using the SpectraMax Paradigm Multi-Mode Detection Platform (Molecular devices LLC, CA, USA). All experiments were carried out 4 times in duplicates. Fluorescence intensity, which is directly proportional to the concentration of surviving parasites, was converted to percentage survival. Cytotoxic concentrations at 50% (CC_50_) were eventually obtained by sigmoidal dose–response curve analysis using the scatter plot option of Microsoft Excel 2016 and expressed as mean of samples ± standard deviation (SD). The selectivity index (SI) was calculated as the ratio of the CC_50_ obtained for both RAW 264.7 and J774 macrophage cells and the IC_50_ for *Leishmania donovani* D10 amastigotes.

### *In vitro* leishmanicidal activity against promastigotes

The effect of N-251 was also evaluated in logarithmic phase promastigotes of the *L*. *donovani* complex using the Invitrogen alamar blue assay kit. The experimental procedure carried out was essentially the same as described above. However, 5 × 10^4^ promastigotes were seeded per well in this case. Treated and untreated promastigotes were incubated at 25 °C. Miltefosine was used a reference compound. IC_50_ values were also obtained as described above.

### Anti-leishmanial efficacy of N-251 *in vivo*

To evaluate the anti-leishmanial efficacy of N-251, *L*. *donovani* D10-infected mice, randomly allocated into experimental groups of 5 animals each, were treated orally with 68 mg/kg body weight of N-251 in olive oil. This dose was determined as the maximum concentration at which no toxicity (ruffled fur, severe weight loss, reduced activity and death) was observed in previous antimalarial studies [21]. Miltefosine (10 mg/kg body weight) and olive oil were used as positive and negative controls, respectively. First, 6-week-old BALB/cA mice (CLEA Japan, Inc. Tokyo, Japan) were infected intraperitoneally with 1x10^8^ stationary phase promastigotes. Four weeks post-infection, treatment was administered through a feeding gavage at 12-hour intervals for 14 consecutive days. Initially, treatment efficacy was expressed as Leishman-Donovan Units (LDU), which was determined by sterilely harvesting and weighing the spleen and liver of euthanized mice. The macrophages in the spleen and liver were imprinted on glass slides and then fixed with methanol for 10 minutes. Imprints were then stained with 5% Giemsa in PBS for 25 mins and examined microscopically. LDU was calculated based on the formula: [(Number of *Leishmania* amastigotes per 1000 macrophage cells) x organ weight (g)] [28–33]. Percentage reduction was also calculated as follows: 100-[(LDU of treatment group/LDU of untreated group) x 100] Determination of LDU was done before treatment at 1-day post-treatment.

To further evaluate the anti-leishmanial efficacy of N-251, post-treatment parasite burden levels were also assessed by limiting dilution analysis, modified for *L*. *donovani* in our laboratory. Briefly, portions of harvested mice liver and spleen were cut, weighed and homogenized in tissue grinders sterilely. The homogenate was suspended in a final volume of 2 ml of M-199 complete medium supplemented with 10% Hi-FBS and 1% penicillin/streptomycin. Five-fold serial dilutions (ranging from 1 to 6.5 x 10^−12^) of the homogenate were made in M-199 complete medium plated (100 ul/well) in 96-well flat-bottom tissue culture plates. Plates were stored in a humidified incubator at 25°C for 14 days after which wells were visually examined for growth with an inverted microscope. The presence or absence of motile promastigotes was recorded in each well. The final titer was the last dilution for which the well contained at least one parasite. The number of parasites per gram organ (parasite burden) was calculated as follows: parasite burden = (geometric mean of reciprocal titers from each duplicate/ weight of homogenized cross section) x 20, where 20 is the reciprocal fraction of the homogenized organ inoculated into the first well. Percentage reduction was also calculated as follows: 100-[(parasite burden in treatment group/ parasite burden of control group) x 100]. LDU and limiting dilution results were analyzed using Microsoft Excel 2016 are expressed as mean ± stard error of the mean (SEM) from 5 mice per group. Comparison of means was done using two-tailed Mann-Whitney U-test and differences were considered significant when p ≤ 0.05.

**Fig 1:**
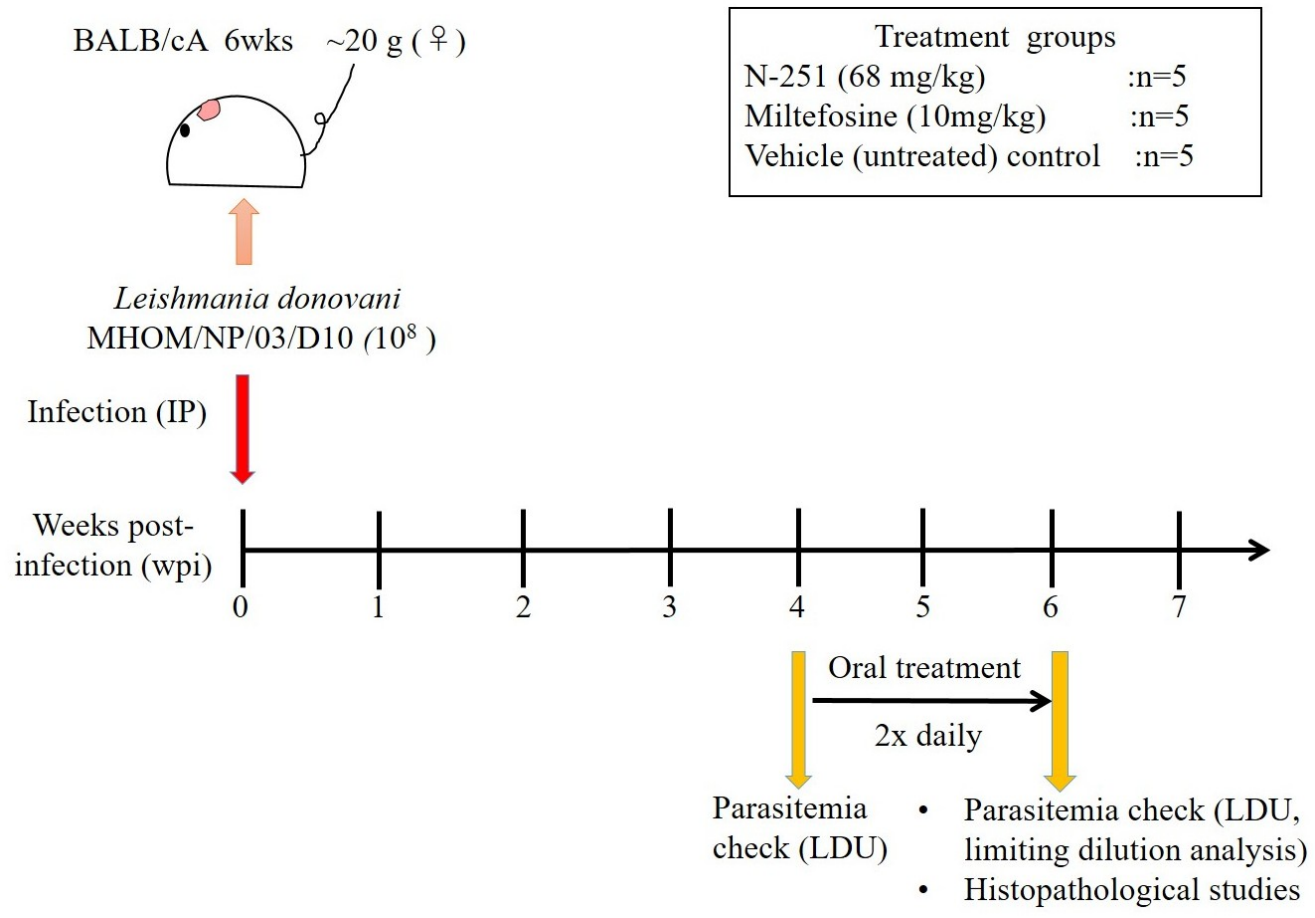
Experimental plan to evaluate the leishmanicidal effect of N-251 in mice.

In addition to assessing post-treatment parasite burden levels, samples of harvested mice liver and spleen were also collected for histological studies. Tissue samples were fixed with formaldehyde for 48 hours and gradually dehydrated with increasing concentrations of ethanol. The tissues were then embedded in paraffin 5 μm thick and sections were cut using a microtome. Thin tissue sections on glass slides were stained with Hematoxylin and Eosin (HE) and analyzed by visualization under light microscope. The number of granulomas was determined with quantification of twenty microscope optic fields using a 40x objective of a light microscope. The experimental plan to evaluate the anti-leishmanial efficacy of N-251 has been summarized in Fig 1

## Results

### N-251 selectively exhibits leishmanicidal activity against *L*. *donovani* D10 amastigotes

**Fig 2:**
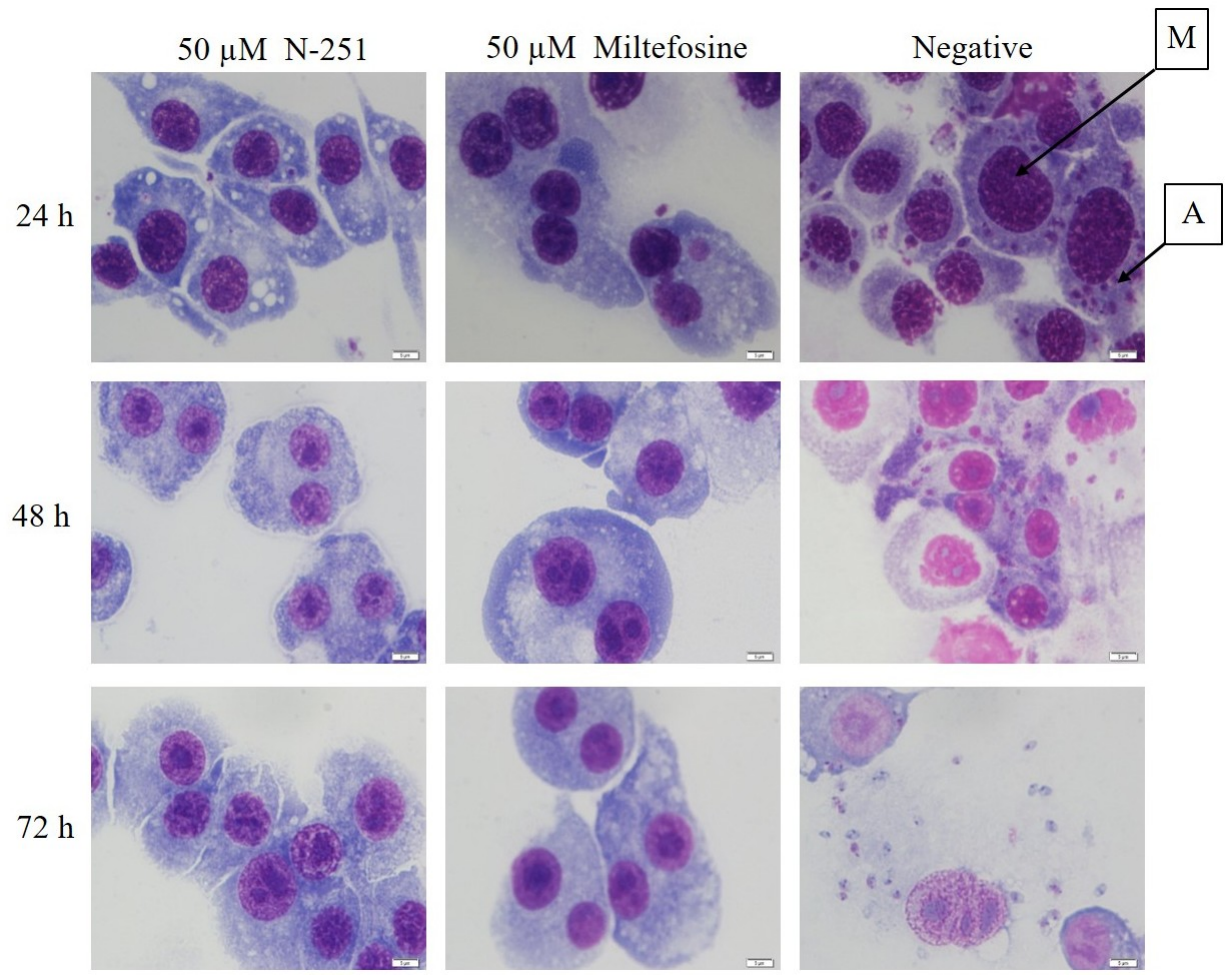
Inhibition of intracellular amastigotes by N-251. Representative Giemsa-stained images (Bar- 20 μm) showing the leishmanicidal effect of N-251 on *L*. *donovani* D10 intracellular amastigotes **(A)** within RAW 264.7 macrophages **(M)** at 24, 48 and 72 hours.

To evaluate the leishmanicidal activity of N-251, we used the amastigote form of *L*. *donovani* D10 parasites as the model parasite because the intracellular amastigote, which lives within the host macrophage, is the form of the parasite responsible for clinical symptoms of VL observed in the human host. Therefore, targeting this parasite form *in vitro* provides a more direct insight into the effect of N-251 *in vivo*. Stationary phase promastigotes and RAW 264.7 macrophages were incubated for 6 h to allow the invasion and transformation of promastigotes within the macrophages. Intracellular amastigotes were then challenged with 0–50 μM of N-251 for 24, 48 and 72 h to investigate the dose-dependent leishmanicidal effect of N-251. In addition, the IC_50_ of N-251 was determined at 72 h by microscopy. Our results clearly show the leishmanicidal effect of N-251 on intracellular amastigotes. When treated with varying concentrations of N-251, *L*. *donovani* amastigotes were eliminated in a dose dependent manner (S2 and S3 Figs) at an IC_50_ of 6.69 ± 0.82, (Table 1). Although most parasites had been cleared by 24 hours, a few macrophages were observed with about one or two intracellular parasites that were probably dead. By 48 and 72 hours, intracellular parasites were completely cleared from all macrophages (Fig 2). The leishmanicidal activity of N-251 was comparable to that of miltefosine (reference drug control). The cytotoxicity of N-251 was evaluated on both RAW 264.7 and J774 murine macrophage cell lines with an alamar blue assay kit for 72 hours. The toxicity of the N-251 treatments was very low among both RAW 264.7 and J774 cells; within the concentration ranges tested, the IC_50_ values were 66.41 ± 4.15 μM and 138.25 ± 24.27 μM, respectively (Table 1). These IC_50_ values correspond to SI values of ≥10 for N-251 (Table 1), suggesting that N-251 is highly specific in its activity against *L*. *donovani* amastigotes, as biological efficacy cannot be attributed to cytotoxicity when the selectivity index is ≥10. The SI values of miltefosine were about twice that of N-251, which were quite comparable (Table 1). The results therefore suggest that N-251 selectively and specifically exhibits leishmanicidal activity by targeting *L*. *donovani* intracellular amastigotes within host macrophage cells, without having any cytotoxic effect on host cells.

**Table 1:** N-251 selectively exhibits leishmanicidal activity against *L*. *donovani* D10 intracellular amastigotes.

Compound	Activity (IC_50_)^±^ μM	Toxicity (CC_50_)* μM		SI^¶^	
	*L*. *donovani* D10	RAW 264.7 macrophages	J774 macrophages	RAW 264.7 macrophages	J774 macrophages
N-251	6.69 ± 0.82^≠^	66.41 ± 4.15	138.25 ± 24.27	9.93	20.67
Miltefosine	1.77 ± 0.05	55.85 ± 5.83	155.75 ± 19.68	31.55	87.99

^**¶**^ Selectivity Index (CC_50_/IC_50_)

^**±**^Concentration at which the inhibition of 50% parasites was observed

*Concentration at which the cytotoxicity of 50% cells was observed

^≠^Values are expressed as the mean ± SD from at least three independent experiments conducted in duplicates.

### N-251 exhibits leishmanicidal activity against various *L*. *donovani* complex parasites

To examine the effect of N-251 on different *L*. *donovani* complex parasites, we screened N-251 against the promastigotes of five parasites belonging to the *L*. *donovani* complex. The parasites used in this study were selected specifically from different geographical locations known to be highly endemic for VL. They include: *L*. *chagasi* PP75 (Brazil), *L*. *donovani* D10 (Nepal) [34,35], *L*. *donovani* DD8 (India), *L*. *donovani* KH (ATCC 30503) (Sudan) and *L*. *infantum* EP173 (Turkey). Our results clearly show the leishmanicidal effect of N-251 on various parasites of the *L*. *donovani* complex. When treated with N-251, leishmanicidal activity was observed against all three *L*. *donovani* (D10, Dd8 KH) parasites, as well as *L*. *chagasi* and *L*. *infantum*, in a dose dependent manner (S4 Fig). The IC_50_ values obtained ranged from 6.12 ± 1.64 μM to 26.90 ± 2.51 μM (Table 2). The results shows that the activity of N-251 is reproducible in promastigotes of different *L*. *donovani* complex parasites including *L*. *donovani* D10, which had its amastigote forms eliminated by N-251 in our *in vitro* infection assays. This suggests that N-251 will probably exhibit leishmanicidal activity against their respective amastigote forms, regardless of their geographic origin.

**Table 2:** N-251 exhibits leishmanicidal activity against different *L*. *donovani* complex parasites.

	IC_50_* (μM)	
Parasites	N-251	Miltefosine
*L*. *chagasi* MHOM/BR/74/PP75	23.12 ± 3.06^¶^	6.59 ± 1.45
*L*. *donovani* MHOM/SU/43/KH	22.95 ± 0.35	5.57 ± 0.11
*L*. *donovani* MHOM/NP/03/D10	26.89 ± 1.98	5.9 ± 1.86
*L*. *donovani* MHOM/IN/80/DD8	26.90 ± 2.51	9.955 ± 2.62
*L*. *infantum* EP-173	6.12 ± 1.64	4.35 ± 0.54

*Concentration at which 50% growth inhibition was observed

^¶^Values are expressed as the mean ± SD from four independent experiments conducted in duplicates.

### Therapeutic effect of N-251 in *L*. *donovani* D10-infected BALB/cA mice

**Fig 3:**
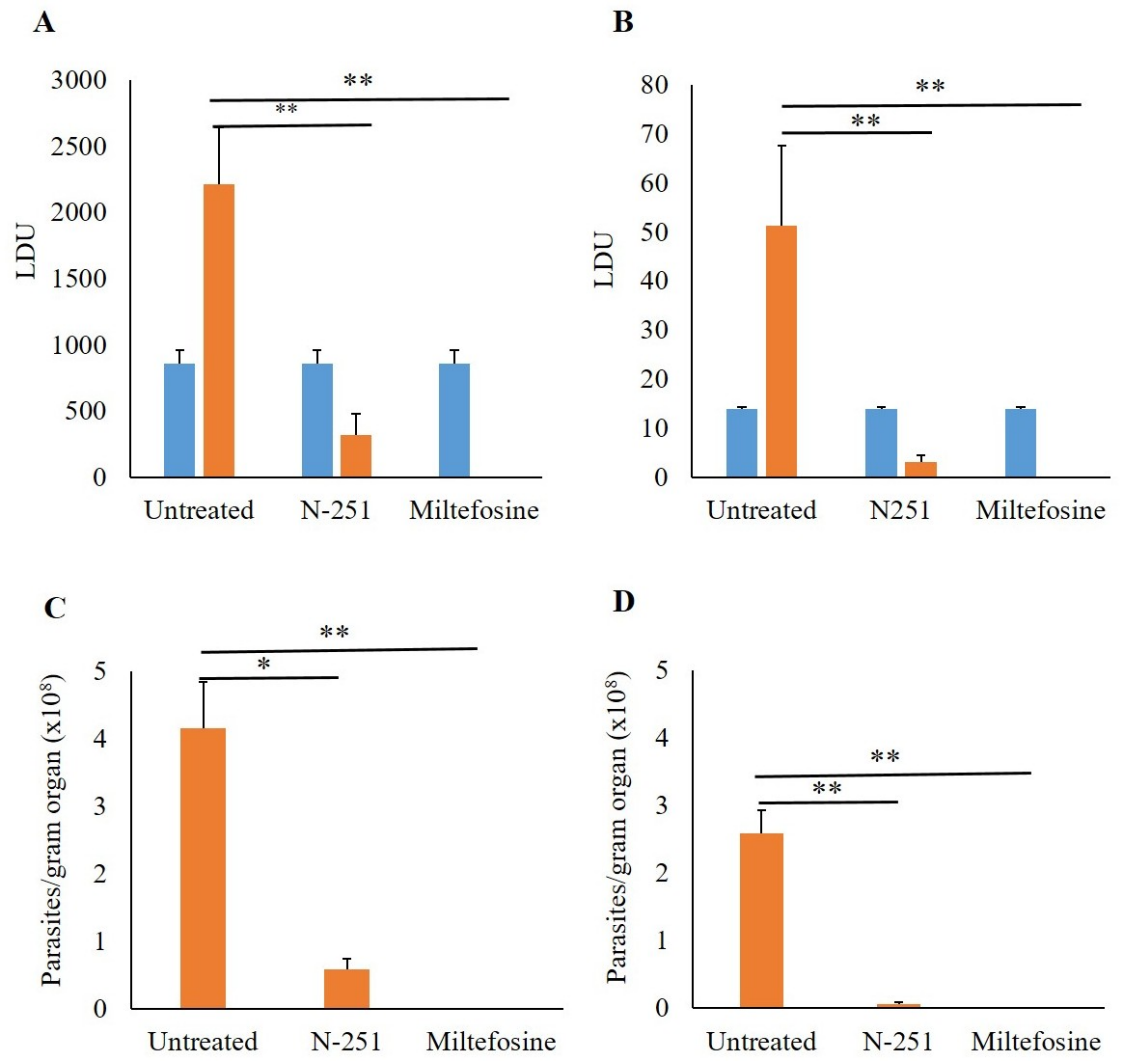
Therapeutic effect of N-251 in *L*. *donovani* D10-infected BALB/cA mice. At 4 wpi, 5 mice were euthanized, followed by the determination of parasite burden in spleen and liver by LDU. The remaining mice were placed into 3 groups (N-251, Miltefosine and vehicle (untreated) control) consisting of 5 mice per group treated orally at 12-hour intervals for 14 days. After treatment (6 wpi), mice were euthanized and parasite burden was determined in spleen and liver tissues by LDU and limiting dilution analyses. Results are represented by bar graphs and are expressed as mean ± SEM from 5 mice in each group. (A) Liver LDU (B) Spleen LDU (C) Liver (Limiting dilution analysis) (D) Spleen (Limiting dilution analysis). Blue: 4 wpi. Orange: 6 wpi. Mean values were compared with the untreated group (6 wpi) and *P ≤ 0.05, **P ≤ 0.01 were considered significant.

The therapeutic efficacy of N-251 was evaluated in *L*. *donovani*-infected mice. Briefly, mice were initially infected intraperitoneally with 10^8^ stationary phase promastigotes, followed by a 4-week incubation period. During this time, parasites invaded the neutrophils and mononuclear phagocytes within the spleen and liver, and a chronic infection was established [28,36,37]. Mice were then treated orally through feeding gavages at 12-hour intervals for 14 days. LDU was then determined in harvested spleen liver tissues before and after treatment. Before treatment, LDU in the liver and spleen were 857.73 ± 104.05 and 13.94 ± 0.30, respectively. After treatment, in the vehicle control group that did not receive any drug, LDU increased by over 100% to 2214.34 ± 426.73 in the liver and 51.38 ± 16.24 in the spleen. In contrast, in N-251-treated mice, LDU decreased significantly (p = 0.012) by 85.73% and 93.98% to 316.00 ± 161.14 and 3.09 ± 1.29 within the liver and spleen, respectively, relative to the untreated group at 6 weeks post-infection (wpi). These results clearly show that after 14 days of treatment, parasites were significantly cleared by N-251(Fig 3a and 3b). Similarly, in miltefosine-treated mice, parasites were cleared at levels that were comparable to those obtained by N-251.

To further confirm the therapeutic effect of N-251, we also evaluated its efficacy by limited dilution analysis to determine parasite burden levels post-treatment. Results here also showed that N-251 significantly cleared *L*. *donovani* parasites. After treatment, parasite burden in the untreated group were determined at 4.15 x 10^8^ and 2.59 x 10^8^ parasites/g organ in liver and spleen respectively. However, in N-251-treated mice, parasite burden decreased significantly (p < 0.05) by 85.89% and 97.41% to 5.86 x 10^7^ and 6.68 x 10^6^ parasites/g organ in liver and spleen respectively (Fig 3c and 3d). *In vivo* data obtained by both LDU and limiting dilution analyses therefore support our *in vitro* observation that N-251 has leishmanicidal activity against *L*. *donovani* amastigotes. Data herein therefore demonstrates the therapeutic effect of N-251 as an oral drug in *L*. *donovani*-infected mice.

**Fig 4:**
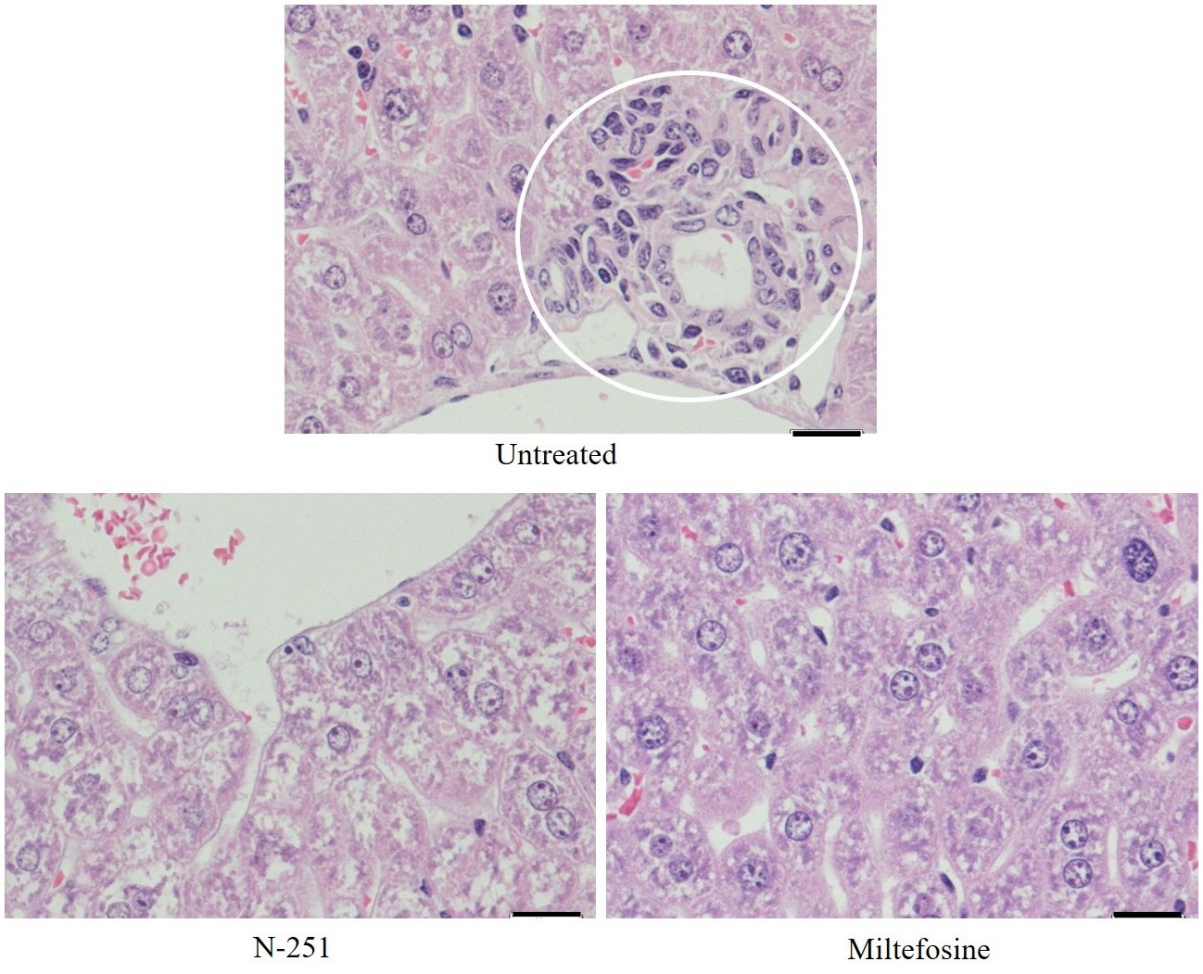
Histological analysis of N-251-treated *L*. *donovani*-infected BALB/cA mice liver tissues. Samples of compound-treated and untreated mice spleen and liver tissues were collected for histological examinations. Five μm thick paraffin tissue sections on glass slides were stained with Hematoxylin and Eosin (HE) analyzed under a light microscope. The results are indicated by representative images obtained from the light microscope. White circle: granuloma formation, Scale bars: 5 μm.

One of the major signs of histopathological damage known to be associated with *L donovani* infection in mice humans are granuloma formation in the liver [38]. Hence, we investigated efficacy of N-251 in suppressing tissue damage by VL. To accomplish this, samples of compound-treated untreated mice liver tissues were collected and processed for histological examinations. The results clearly showed that N-251 is able to suppress damage due to granuloma formation in *L*. *donovani*-infected liver. The untreated group revealed high levels of granuloma formation (an average of about 4 per field) within mice liver. In contrast, when mice were treated with N-251, no granuloma formation was observed within the liver (Fig 4). These results demonstrate that N-251 significantly suppresses and improves the conditions of *L*. *donovani*-infected mice liver tissues as a result of significant parasite reduction.

## Discussion

The demand for novel, orally administered alternatives to miltefosine for the treatment of VL has become more urgent. This is particularly true as existing drugs are plagued with several limitations, such as their invasive means of administration and long duration regimens [39]. Currently, miltefosine is the sole orally-administered drug for the treatment of VL. Recently, the widespread use of miltefosine, coupled with recent reports of increasing treatment relapses, have raised concerns of possibility of the rapid emergence of resistance due to its long half-life [40,41], thus, highlighting the urgent need for novel, oral antileishmanial drugs. In the present study, we showed that N-251, a novel, synthetic, orally-administered endoperoxide, selectively and specifically kills *L*. *donovani* amastigotes with no toxicity to host cells. Even after a progressive infection was established in mice, N-251 eliminated intracellular amastigotes, resulting in the suppression of hyper-granuloma formation in mice liver. The formation of hepatic hyper-granulomas is known to be mostly elicited by the presence of intracellular amastigotes in *Leishmania*-infected hosts [38,42,43]. This therefore suggest that the significant reduction of intracellular amastigotes by N-251 observed in this study, may have resulted in the absence of granuloma, indicating the potential of N-251 as a lead compound for the development of an oral chemotherapy against VL.

The cleavage of the endoperoxide bridge (C-O-O-C, S1 Fig) generates short-lived, cytotoxic oxyradicals in the presence of heme iron or free Fe^2+^. Fenton degradation of the oxyradical intermediates can produce hydroxyl radicals (OH) that are highly reactive against a wide variety of molecules such as amino acids, enzymes, lipids and nucleic acids [44,45]. The released radicals then oxidize these molecules, thereby inhibiting their functions, which eventually leads to parasite death. This explains why endoperoxides exhibit a wide spectrum of anti-infective activity. N-251 is also characterized by a peroxide bridge within its structure that can be cleaved and activated in the presence of a heme iron or free Fe^2+^ [20,46,47], suggesting that this is the basis for its antileishmanial activity observed in both promastigotes and amastigotes screened in this study. *Leishmania* are known to forage for iron from host macrophages for their growth for defense against the macrophage’s oxidative assault by providing iron to the antioxidant enzyme superoxide dismutase [19,48,49]. The accumulation of iron within the parasite therefore results in the selective killing of the parasite by N-251. This also explains the 4-fold increase in activity observed against intracellular amastigotes relative to that of promastigotes.

Some of the current antileishmanial drugs, including pentavalent antimonials and paromomycin, have been reported to exhibit a limited spectrum of antileishmanial activity, which is mostly dependent on the species of *Leishmania*, the geographical location, as well as the clinical presentation of the disease [50–54]. However, in this study, N-251 exhibited leishmanicidal effects against various *L donovani* complex parasites from different parts of the world. This therefore shows the reproducibility of N-251’s activity in different *L*. *donovani* complex parasites, suggesting that N-251 may exhibit leishmanicidal activity against different VL parasites, regardless of their geographical origin. In addition, in previous studies, N-251 was observed to be active against other non-*Leishmania* parasites such as *Plasmodium*, *T*. *gondii* and *Schistosoma* [20,21,23,47,55]. It is therefore quite clear that N-251 exhibits a wide spectrum of activity. In general, advantages of wide spectrum compounds include cost effectiveness in using one drug to treat different infectious diseases as well as serving as good options for Mass drug administration exercises in poor endemic regions [56,57]. This inherent characteristic of N-251 appears to be one of the several advantages it has over other existing antileishmanial drugs.

Miltefosine, the only oral drug currently available against VL, affects parasites by the disruption of parasitic Ca^2+^ homeostasis via opening of the sphingosine-activated plasma membrane Ca^2+^ channel, together with the impairment of the acidocalcisomes [58]. However, it is limited by its teratogenicity and several side effects. There are also concerns that its use as a monotherapy can eventually lead to the rapid emergence of resistant parasites [40,59]. In fact, treatment relapses among VL patients [60], as well as the identification of miltefosine-resistant clinical strains, have already been reported [61,62]. Our compound, N-251, is also orally-administered and acts through a different mode of action from miltefosine, making it a good candidate for possible combination with miltefosine. According to the WHO, combination therapy is an important strategy to improve leishmaniasis therapy and also delay the emergence of resistance [63,64], as can be seen in the case of malaria, tuberculosis, and HIV [65–67]. In future studies, we will therefore evaluate the combinatory effect of N-251 and miltefosine in experimental VL.

In conclusion, results herein demonstrates the *in vitro* and *in vivo* antileishmanial effect of the novel orally administered synthetic endoperoxide, N-251. Also, the broad-spectrum activity of N-251 against various parasites of *L*. *donovani* complex from different geographical locations was established. More importantly, this study highlights the importance of N-251 as an oral drug for monotherapy its possible combination with miltefosine. Finally, N-251 may be a promising lead compound for the development of new oral chemotherapy against visceral leishmaniasis.

## Supporting information

S1 Fig Chemical Structure of novel synthetic endoperoxide, 6-(1,2,6,7 -tetraoxaspiro[7.11]nonadec-4- yl)hexan-1-ol (N-251). (TIF)

S2 Fig Dose-dependent leishmanicidal effect of N-251 on intracellular amastigotes. Representative Giemsa-stained images (Scale bar-5 μm) showing the dose-dependent effect of N-251 against *L*. *donovani* D10 amastigotes within RAW 264.7 macrophages.
(TIF)

S3 Fig Dose–response curves showing the effect of N-251 on intracellular amastigotes. (TIF)

S4 Fig Dose-dependent leishmanicidal activity of N-251 against *L*. *donovani* complex promastigotes. Dose–response curves showing the dose-dependent effect of N-251 against *L*. *donovani* complex promastigotes and macrophage cells.
(TIF)
