# Supplementary figures and images for: Oral activity of the antimalarial endoperoxide 6-(1,2,6,7-tetraoxaspiro[7.11]nonadec-4-yl)hexan-1-ol (N-251) against Leishmania donovani complex
Source: PLoS Negl Trop Dis. 2019 Mar 25;13(3):e0007235. doi: 10.1371/journal.pntd.0007235 (PMC6433226; doi:10.1371/journal.pntd.0007235)

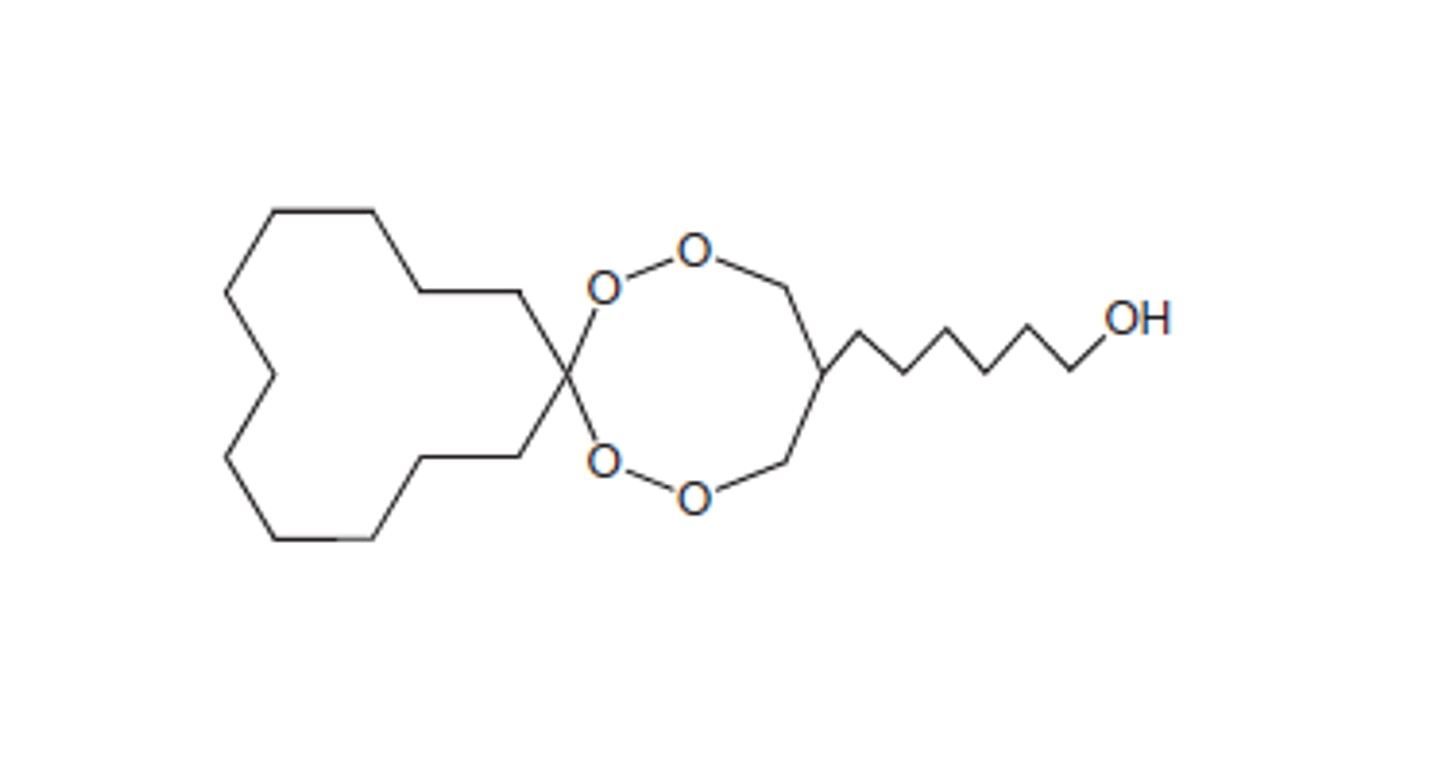

Supplement: S1 Fig — (TIF) [file pntd.0007235.s001.tif]

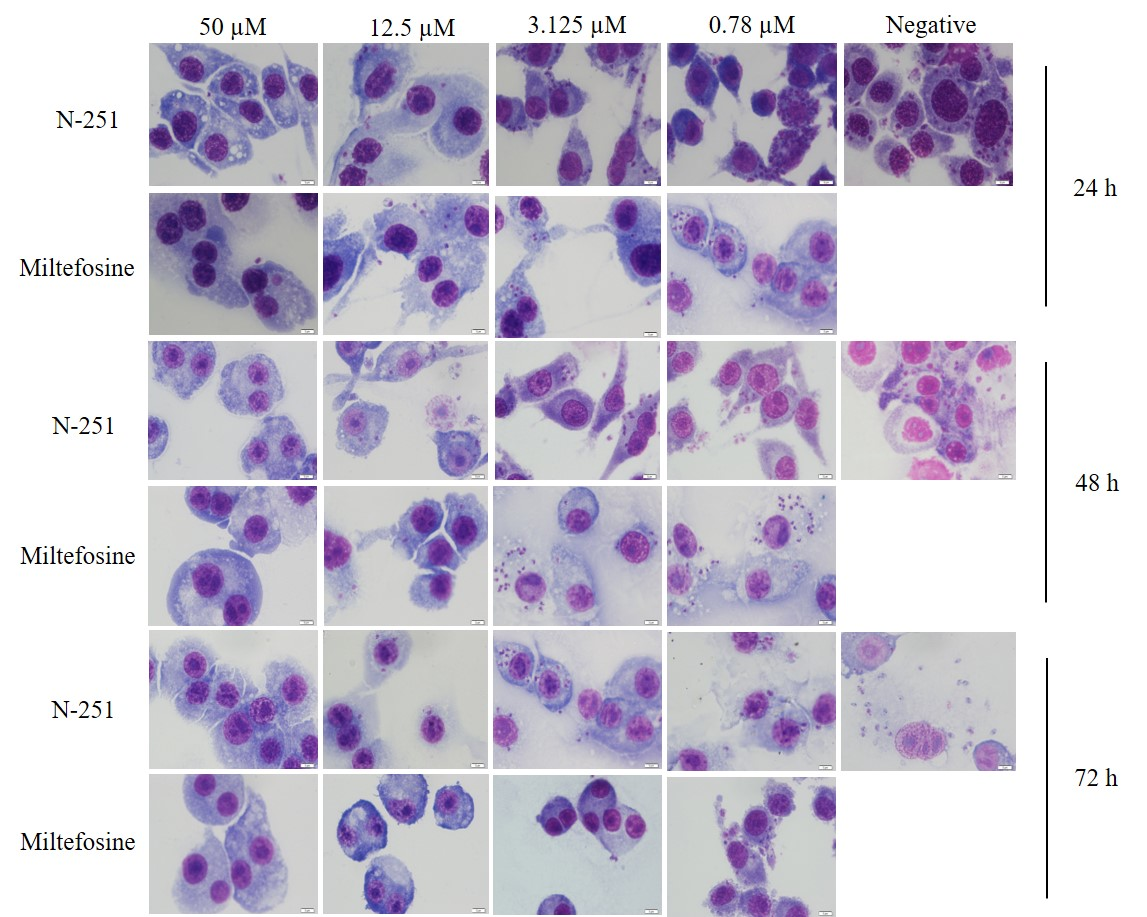

Supplement: S2 Fig — Representative Giemsa-stained images (Scale bar-5 μm) showing the dose-dependent effect of N-251 against L. donovani D10 amastigotes within RAW 264.7 macrophages. (TIF) [file pntd.0007235.s002.tif]

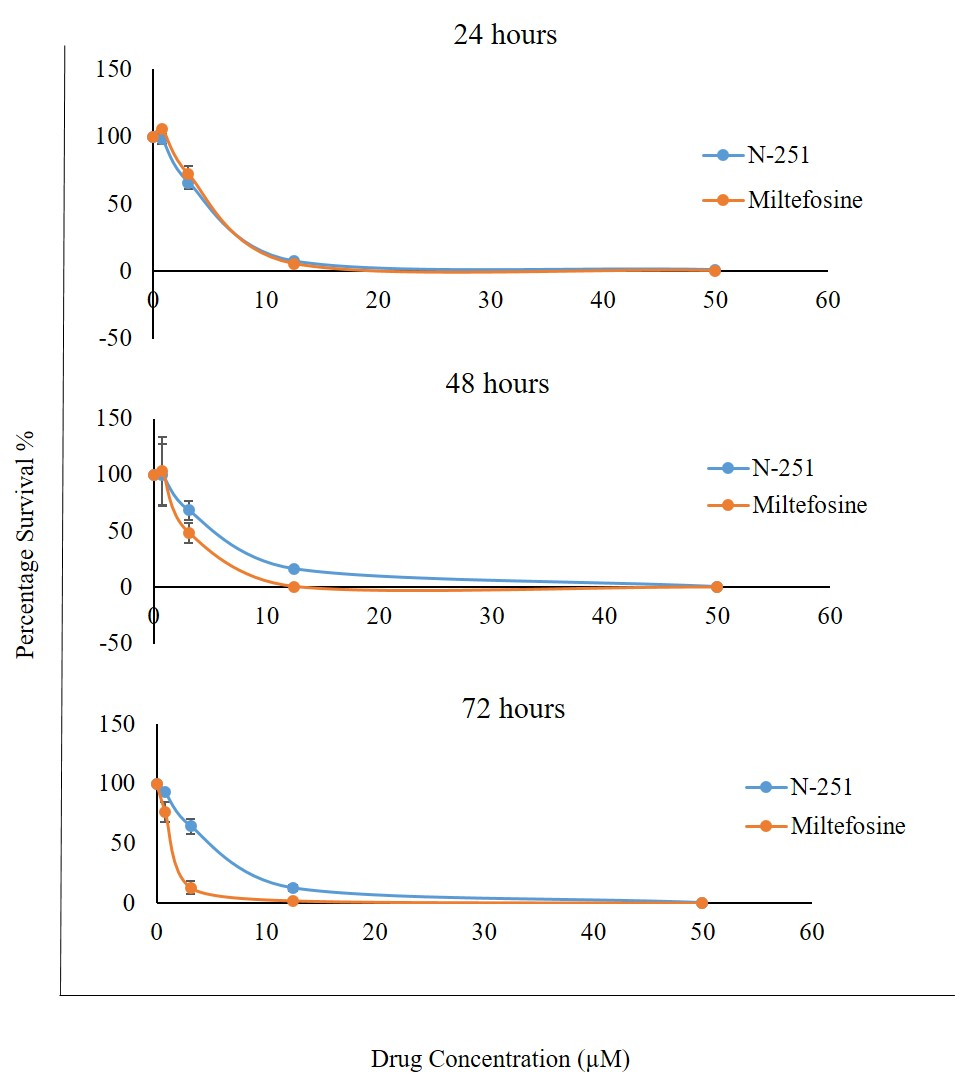

Supplement: S3 Fig — (TIF) [file pntd.0007235.s003.tif]

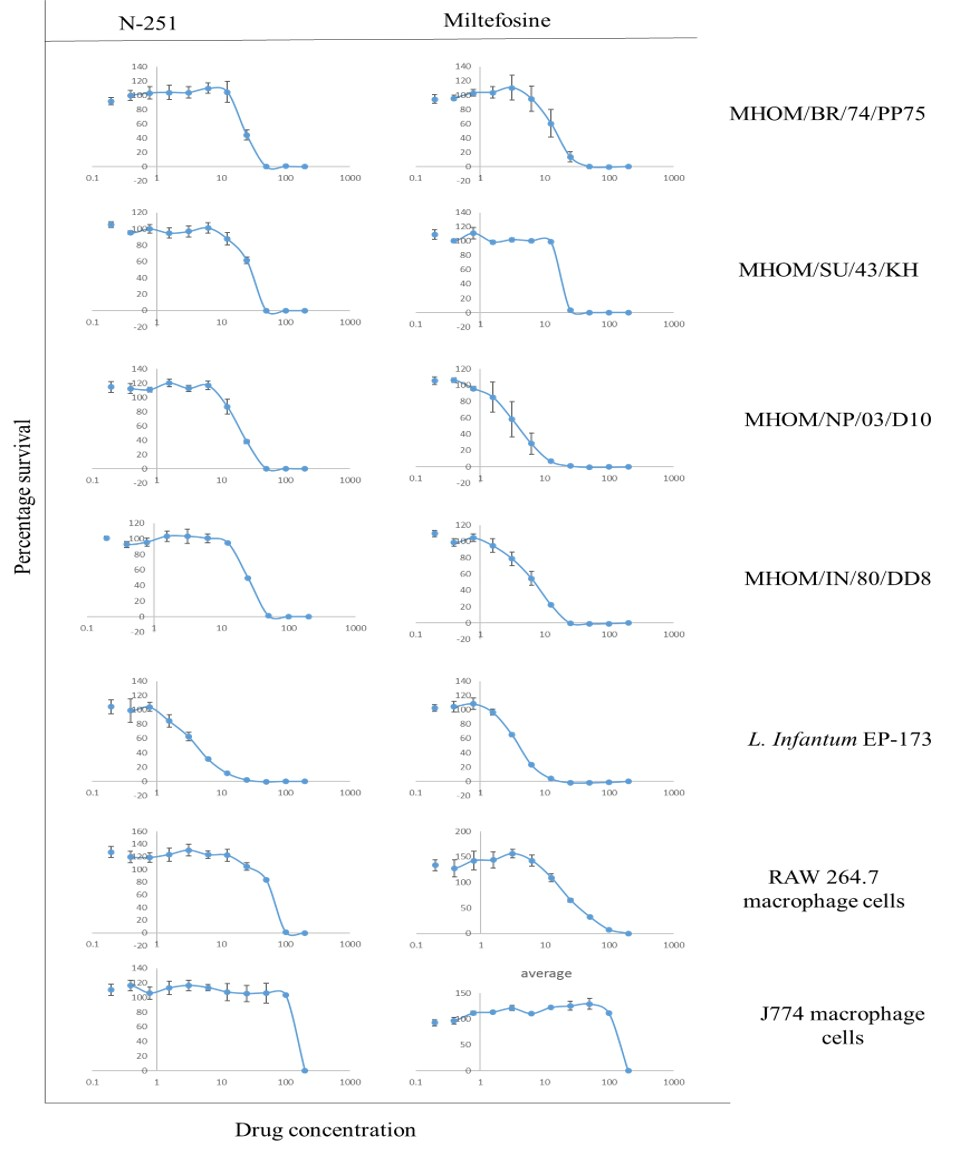

Supplement: S4 Fig — Dose–response curves showing the dose-dependent effect of N-251 against L. donovani complex promastigotes and macrophage cells. (TIF) [file pntd.0007235.s004.tif]
